# Reply to: Best BLAST hit alone cannot be used as evidence of fraud

**DOI:** 10.1038/s41598-022-26737-3

**Published:** 2023-01-17

**Authors:** Carmen Blanco-Fernandez, Alba Ardura, Gonzalo Machado-Schiaffino, Eva Garcia-Vazquez

**Affiliations:** Department of Functional Biology, University of Oviedo, 33006 Oviedo, Spain

**Keywords:** Genetics, Biological techniques

**replying to**: N. Diaz-Arce; *Scientific Reports* 10.1038/s41598-022-26720-y (2023).

**Figure 1:**
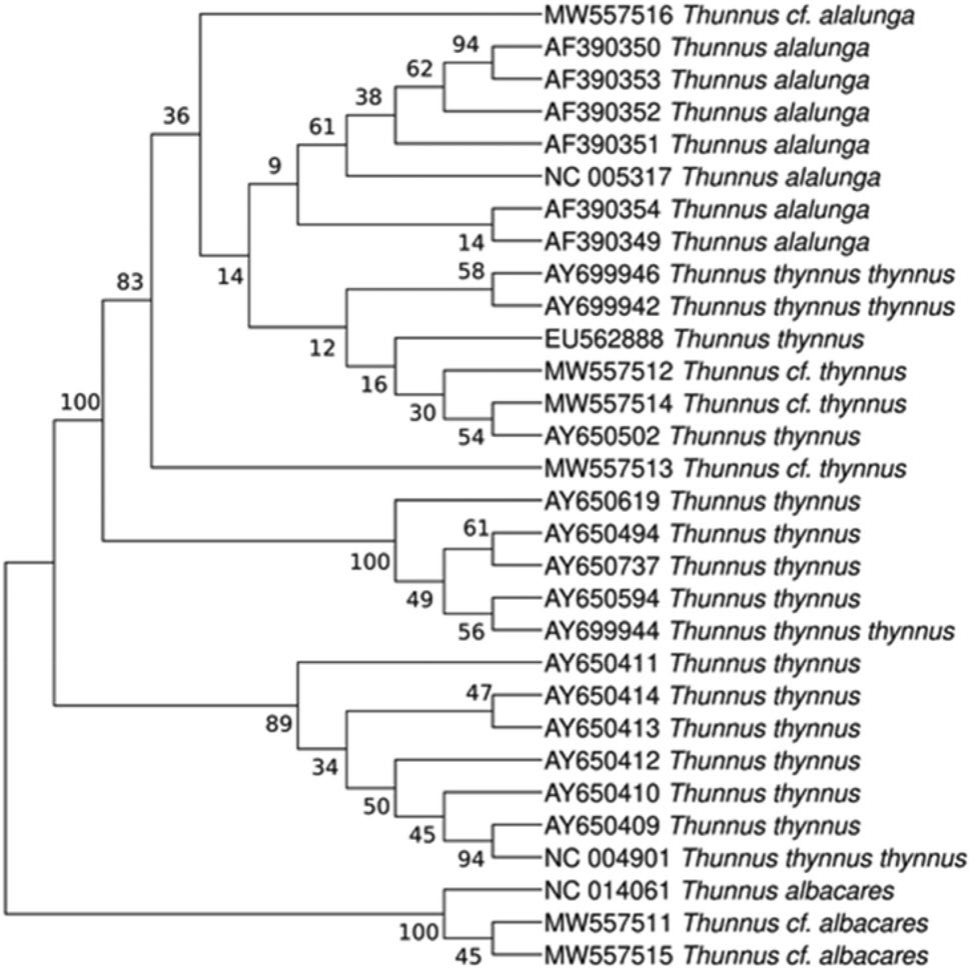
Maximum Likelihood tree built from problem sequences from Blanco-Fernandez et al.^2^ (MW557511-16) and reference tuna sequences from GenBank.

Diaz-Arce and Rodriguez-Ezpeleta^1^ discuss the utility of barcoding using best-match criterion for the identification of fish species from commercial samples. Focusing on tuna, the evidence provided is a phylogenetic tree containing six problem mitochondrial control region sequences of Blanco-Fernandez et al.^2^ and 21 reference sequences taken from databases: 8 *Thunnus thynnus*, 5T*. thynnus* with *T. alalunga* introgression, 7T*. alalunga*, 1T*. albacares*. Three problem sequences (MW557512, MW557513 and MW557514) identified as *T. thynnus* from best BLAST hit^2^ clustered together with the *T. alalunga* references and only one *T. thynnus* reference, while the rest of *T. thynnus* were in other clades. This tree poses doubts about the species status of those three samples barcoded as *T. thynnus*, questioning the conclusions obtained from barcoding. However, with a few references per species it is very difficult to capture the variation of the control region in *T. thynnus* and *T. alalunga*. The results can vary depending on the references selected from GenBank to reconstruct the tree. As a proof of this we produced a tree adding three more references to the sequences employed by Diaz-Arce and Rodriguez-Ezpeleta^1^, with the same parameters and testing the best-fit evolutionary model. In the resulting tree (Fig. 1) the cluster that contains the three problem sequences has now four *T. thynnus* references: AY650502, AY699942, AY699946 and EU562888 (Table 1). AY650502 (haplotype BFT94 in Alvarado Bremer et al.^3^) comes from a *T. thynnus* voucher specimen identified as introgressed with *T. alalunga*^3^. AY699942 and AY99946 correspond to *T. thynnus* sampled for population genetics^4,5^. EU562888 belongs to an individual morphologically identified as *T. thynnus* used in a study of population genetics in this species^6^. From this tree the problem sequences MW557512, MW557513 and MW557514, assigned to *T. thynnus* from barcoding, can be interpreted as belonging to introgressed *T. thynnus*. Alternatively, we could treat them as *T. alalunga*, although the best match in BLAST was *T. thynnus*^6^ in the three cases^2^.

BLAST-based barcoding with mitochondrial markers has been employed alone to identify hakes, monkfish, tunas, catfish, and many other fish species using best-match criterion^7–10^. However, the reasonable doubt that arises from Fig. 1 cannot be solved without nuclear markers. Phylogenetic studies show *T. thynnus* has haplotypes corresponding to *T. alalunga* mtDNA^3^, while the reciprocal introgression of *T. thynnus* mitochondrial DNA in albacore has not been detected^3,11^. Nuclear markers such as the first internal transcribed spacer (ITS) within the nuclear rDNA could be employed to solve this ambiguity. This marker has been already used to distinguish between *T. alalunga* and *T. thynnus*^12^, and would allow to confirming *T. thynnus* issued from former hybridization events involving *T. alalunga* females.

**Table 1:** Accession numbers and species as they appear in GenBank for the sequences employed to reconstruct the ML tree of Fig. 1.

Accession Nr.	GenBank species	Accession Nr	GenBank species
MW557516.1	*Thunnus cf. alalunga*	AY650619.1	*Thunnus thynnus*
AF390350.1	*Thunnus alalunga*	AY650494.1	*Thunnus thynnus*
AF390353.1	*Thunnus alalunga*	AY650737.1	*Thunnus thynnus*
AF390352.1	*Thunnus alalunga*	AY650594.1	*Thunnus thynnus*
AF390351.1	*Thunnus alalunga*	AY699944.1	*Thunnus thynnus thynnus*
NC_005317.1	*Thunnus alalunga*	AY650411.1	*Thunnus thynnus*
AF390354.1	*Thunnus alalunga*	AY650414.1	*Thunnus thynnus*
AF390349.1	*Thunnus alalunga*	AY650413.1	*Thunnus thynnus*
AY699946.1	*Thunnus thynnus thynnus*	AY650412.1	*Thunnus thynnus*
AY699942.1	*Thunnus thynnus thynnus*	AY650410.1	*Thunnus thynnus*
EU562888.1	*Thunnus thynnus*	AY650409.1	*Thunnus thynnus*
MW557512.1	*Thunnus cf. thynnus*	NC_004901.2	*Thunnus thynnus thynnus*
MW557514.1	*Thunnus cf. thynnus*	NC_014061.1	*Thunnus albacares*
AY650502.1	*Thunnus thynnus*	MW557511.1	*Thunnus cf. albacares*
MW557513.1	*Thunnus cf. thynnus*	MW557515.1	*Thunnus cf. albacares*

In summary, we recognize that the assignation of problem sequences to *Thunnus thynnus* in Blanco-Fernandez et al.^2^, although supported from barcoding and not rejected from phylogenies, should be validated employing nuclear markers. This can be extended to the rest of cases where interspecific introgression occurs. Finally, we have renamed the problem sequences reported in Blanco-Fernandez et al.^2^ including the Open Nomenclature qualifier *cf.* (= the identification is not achievable without further comparison with reference material^13^) before the species name, stating in their description that they were obtained from seafood samples (see Accession numbers MW557511-MW557516). This way we hope to reduce the noise in databases commented by Diaz-Arce and Rodriguez-Ezpeleta^1^.
